# Acaricidal Activity and Synergistic Effect of Thyme Oil Constituents against Carmine Spider Mite (*Tetranychus Cinnabarinus* (Boisduval))

**DOI:** 10.3390/molecules22111873

**Published:** 2017-11-01

**Authors:** Lipeng Wu, Xin Huo, Xiaolong Zhou, Duoyong Zhao, Weizhong He, Shenghong Liu, Hejiang Liu, Ting Feng, Cheng Wang

**Affiliations:** 1 Institute of Quality Standards & Testing Technology for Agro-Products, Xinjiang Academy of Agricultural Sciences, Urumqi 830091, Xinjiang, China; wlp@nwafu.edu.cn (L.W.); zxlxaas@sina.com (X.Z.); zdyxaas@sina.com (D.Z.); zjxaas@sina.com (W.H.); stxaas@sina.com (S.L.); wjmxaas@sina.com (H.L.); ftxaas@sina.com (T.F.); 2 Laboratory of Quality and Safety Risk Assessment for Agro-Products (Urumqi), Ministry of Agriculture, Urumqi 830091, Xinjiang, China; 3 Key Laboratory of Agro-Products Quality and Safety of Xinjiang, Urumqi 830091, Xinjiang, China; 4 Agro Technical Extension Center of Altay Prefecture, Altay 836500, Xinjiang, China; xin0564@sina.com

**Keywords:** plant essential oil, thyme oil, thymol, synergistic effect, botanical acaricides

## Abstract

Studies examining the use of essential oils as replacements for synthetic insecticides require an understanding of the contribution of each constituent present, interactions among these components, and how they relate to overall toxicity. In the present study, the chemical composition of commercial thyme oil was identified by gas chromatography-mass spectrometry. Thyme oil and blends of its major constituents were tested for their acaricidal activitities against carmine spider mites (*Tetranychus cinnabarinus (Boisduval)*) using a slide-dip bioassay. Natural thyme oil showed greater toxicity than any single constituent or blend of constituents. Thymol was the most abundant component (34.4%), and also possessed the strongest acaricidal activity compared with other single constituents. When tested individually, four constituents (linalool, terpinene, *p*-cymene and carvacrol) also had activity, while α-pinene, benzoic acid and ethyl gallate had almost no activity. The toxicity of blends of selected constituents indicated a synergistic effect among the putatively active and inactive constituents, with the presence of all constituents necessary to reach the highest toxicity. The results indicated that thyme oil and some of its major constituents have the potential to be developed into botanical acaricides.

## 1. Introduction

Worldwide, an estimated 5% of agricultural production losses are caused by various species of spider mites. The carmine spider mite *Tetranychus cinnabarinus* (Boisduval), in particular, has long been recognized as one of the most notorious of tetranychid mites. This species have been shown to cause serious damage to the quality and yield of many crops, fruits and vegetables grown in the field or in greenhouse all over the world by damaging foliar tissues, reducing photosynthetic activity and even causing leaf abscission when severe infestations occur [1,2]. Traditionally, control of these mites has involved the use of synthetic chemical pesticides [3], however, the intensive use of chemical pesticides, coupled with the high fecundity, short life cycle and arrhenotoky genetics of these mites, have resulted in the rapid evolution of insecticide resistance [4,5]. Specifically, carmine spider mites have been reported to have resistance against a number of commonly used pesticides, including dicofol, omethoate, amitraz, pyridaben and fenpropathrin [6,7]. The frequent use of these non-selective synthetic pesticides has been shown to have adverse effects on both the environment and human health [8,9], and also been linked to mortality of beneficial natural enemies of the mites, resulting in a resurgence of mite populations. These problems with increasing levels of resistance to conventional acaricides have led to applications of more frequent and higher doses of insecticides, which further increase the risk to humans and the environment [10]. Thus, studies examining alternatives to traditional chemical treatments such as botanical acaricides that have lower mammalian and environmental toxicity, are being performed [11].

Active substances of botanical pesticides are secondary plant metabolites synthesized by some plants as a self-defense mechanism to fight against attacks of insect pests and pathogens. Such substances include so-called essential oils, which are complex mixtures of a large number of low molecular weight compounds [12]. The use of essential oils as botanical pesticides has numerous advantages. First of all, complex mixtures of active substances contained in essential oils, with different modes of action and often exhibiting mutual synergistic effects, can slow down the development of resistant pest populations [13]. It has been reported that green peach aphids develop resistance to pure azadirachtin (the major constituent of neem insecticide), but not to a neem seed extract containing not only the same amount of azadirachtin but also many other constituents [14]. In addition, essential oils have low persistence under field conditions because of their volatility, resulting in the low residue level after application. Consequently, they cause less negative effects on environmental and human health. Besides that, most of the essential oils with few exceptions have low toxicity against non-target organisms, including humans, and many of them are permitted to be used in foods and medicines as flavorings [11].

The chemical compositions of essential oils can be influenced by many factors including genotypes, geographic differences, nutrition levels, harvest time and extraction methods [15]. These variations of chemical compositions also lead to different bioactivities. Isman et al. demonstrated that ten different commercial rosemary oils showed 2.2- and 5.7-fold different toxicities against armyworm and cabbage looper, respectively [16]. Understanding the contribution of each individual constitution and their interactions to the overall toxicity is prerequisite to use essential oils as substitutions to synthetic insecticides. As these are important factors that can ensure the quality of botanical pesticides based on essential oils.

Essential oils have been shown to have insecticidal activity against a number of insects including stored product pests [17], cockroaches [18], aphids [19], cabbage looper [20], mosquitoes [21], cigarette beetle [22] and spider mites [23,24,25]. In our previous screening program for acaricidal activity of over fifty commercial essential oils, we found that thyme oil possessed the strongest toxicity against *T. cinnabarinus* (unpublished data). Thyme oil has also been reported to have the insecticidal activity [26], fumigant activity [24], and physiological impact such as feeding deterrence or insect larval growth [27,28] as well as acaricidal activity [29]. Although many screening data have demonstrated that thyme oil can be considered as a promising insect control agent, the activity of each constituent, their contributions to the overall toxicity and the synergistic interactions between the active constituents of thyme oil against carmine spider mite have never been reported.

Therefore, it is worthwhile to do some research on thyme oil in terms of its acaricidal activity against carmine spider mite. The aims of our present work were to analyze the chemical composition, identify the contribution of individual constituents to the overall toxicity and assess the combined toxic effect between the active constituents of thyme oil against carmine spider mite by a slide-dip bioassay.

## 2. Results

### 2.1. Chemical Composition of Thyme Oil

GC-MS analysis identified 12 different compounds, which accounted for 97.8% of the total chemical composition of the thyme oil sample tested (Table 1). Thymol (34.6%) was the most abundant compound, followed by *p*-cymene (30.8%), terpinene (15.6%), linalool (9.4%) and carvacrol (2.5%). The other 7 components, α-pinene (1.4%), ethyl gallate (1.3%), benzoic acid (1.2%), α-thujene (0.3%), camphor (0.3%), camphene (0.2%) and borneol (0.2%) were identified in lesser amounts. A few unknown compounds (2.2%) were found as trace or minor components.

### 2.2. Toxicities of Thyme Oil and Individual Constituents

Bioassays of thyme oil and major single constituents (proportion > 1%) revealed that natural thyme oil possessed greater acaricidal activity than any other single constituent, whether at the same level (2500 mg/L) or at the level based on the proportion of thyme oil (*p* < 0.05; Table 1). Among all major single constituents, one compound (thymol) was the most toxic, while four compounds (linalool, terpinene, *p*-cymene and carvacrol) were slightly but not significantly toxic, and the remaining three compounds (α-pinene, benzoic acid, and ethyl gallate) were non-toxic to mites (*p* < 0.05; Table 1). In order to further compare the toxicity of thyme oil and thymol, toxicity regression lines of thyme oil and thymol were calculated based on the mortality after 24 h of treatment. The results showed that thyme oil was more toxic than thymol with their LC_50_ of 762.1 mg/L and 1183.9 mg/L, respectively (Table 2).

### 2.3. Comparative Toxicities of Individual Constituents and Blends

**Figure 1:**
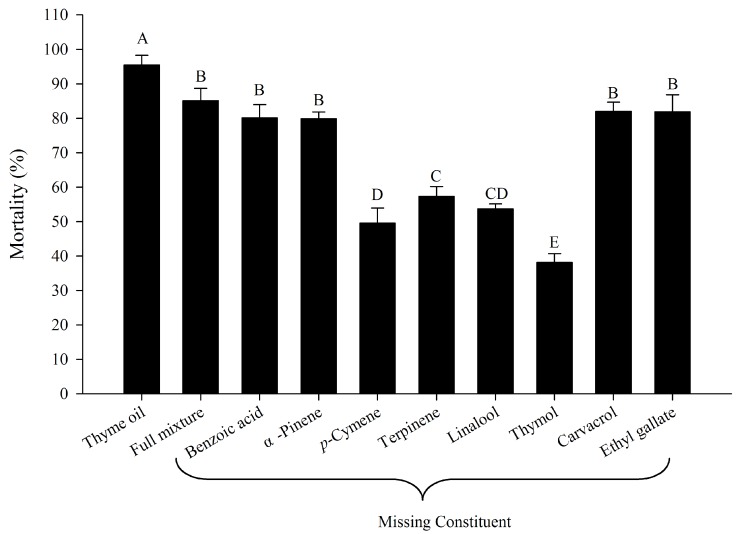
Mortality caused by natural thyme oil, full mixture, and blends of constituents of thyme oil when applied at the concentration at which the natural thyme oil causes 95% mortality. “Full mixture” indicates a blend of eight major constituents (proportion > 1%), whereas all others indicate full mixture missing the constituent noted. Error bars represent the standard error of the mean of four replicates (40 mites per replicate). Means followed by different letters are significantly different from each other (Tukey’s test, *p* < 0.05).

In order to identify the contribution of individual constituents to the total toxicity of thyme oil, we compared the toxicity of natural thyme oil, a complete artificial mixture and incomplete artificial mixtures each lacking one major compound (proportion > 1%). The results showed that there was a significant difference between the mortality caused by natural thyme oil and complete artificial mixture, with mortalities of 95.5% and 85.1%, respectively (*p* < 0.05; Figure 1). Component elimination assays indicated that the absence of thymol from the full artificial mixture caused the most significant decrease of the total toxicity. Removal of terpinene, linalool and *p*-cymene also had a significant effect on the total toxicity, but less significant than thymol. Excluding the other four constituents (pinene, benzoic acid, carvacrol and ethyl gallate) from the mixtures did not significantly affect the total toxicity (*p* < 0.05; Figure 1). Therefore, we can conclude that thymol was the most active constituent and three constituents (terpinene, linalool and *p*-cymene) were moderately active constituents while pinene, benzoic acid, carvacrol and ethyl gallate were inactive constituents.

**Figure 2:**
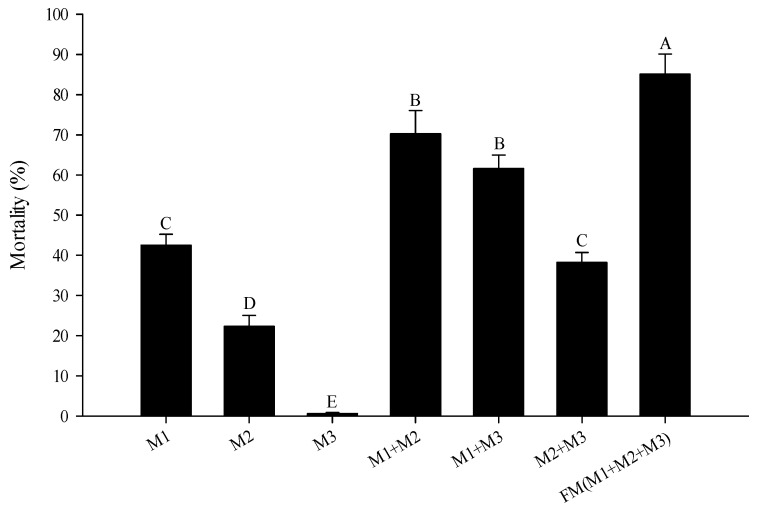
Mortality caused by selected blends of active and inactive constituents of thyme oil when applied at the concentration at which the natural thyme oil causes 95% mortality. M1 (the most active constituent) = thymol; M2 (moderately active constituents) = linalool, terpinene, *p*-cymene and carvacrol; M3 (inactive constituents) = α-pinene, benzoic acid and ethyl gallate. Error bars represent the standard error of the mean of four replicates (40 mites per replicate). Means followed by different letters are significantly different from each other (Tukey’s test, *p* < 0.05).

The comparison of toxicity caused by selected blends of active and inactive constituents (Figure 2) showed that the most active constituent (M1 = thymol) alone caused 42.5% mortality, mixtures of the moderately active constituents (M2 = terpinene, linalool and *p*-cymene) caused 22.3% mortality, mixtures of all active constituents (M1 + M2) caused 70.3% mortality, whereas mixtures of the inactive constituents (M3 = pinene, benzoic acid, carvacrol and ethyl gallate) caused only 0.6% mortality. Combination of the mixtures of M1 + M3 and M2 + M3 caused mortalities of 61.6% and 38.2%, respectively.

### 2.4. Synergistic Toxic Effect of Active Compounds

Binary mixtures of the five active components of thyme oil (thymol, terpinene, linalool, *p*-cymene and carvacrol) were tested for acaricidal activity and were analyzed for any synergistic interactions, using co-toxicity factor (CTF) as a criterion. The observed mortality (OM) of the ten binary mixtures showed statistically significant differences (Chi-square test, x^2^ = 21.37, *p* < 0.05). CTF values for thymol + terpinene and thymol + linalool were greater than 20, indicating a strong synergy. One mixture (linalool + carvacrol) exhibited the antagonistic effect. The other seven combinations with CTF values between −20 and 20 indicated additive effects (Table 3).

## 3. Discussion

### 3.1. Constituents and Toxicity of Thyme Oil

Similar to our results, 16 constituents were identified in commercial thyme oil in a study by Tak et al. [30], with thymol (50.2%) and *p*-cymene (33.1%) of the first and second most abundant compound, respectively. Gaire et al. found that *p*-cymene (29.5%) and thymol (22.7%) were the major components in commercial red thyme oil [31], but there were differences in minor components and the proportion of constituents. Carvacrol accounted for 2.5% of thyme oil in our study but was found as 0 and 1.4% in the study of Tak et al. [30] and Gaire et al. [31], respectively. Lemos et al. proved that the composition and proportion of thyme oil can be remarkably influenced by plant age, collecting seasons and storage time [32]. The variations in composition rate and minor constitutions mentioned above may be attributed to the diversity growing area, different growth time of the plants and different extraction methods used.

Yang et al. tested the toxicity of six commonly used acaricides against carmine spider mite by slide-dip method. The LC_50_ of abamectin (1.8%), emamectin benzoate (1%), pyridaben (15%), spinosad (2.5%) and envidor (240 g/L) were 0.2678, 0.6805, 17.93, 1511 and 1067 mg/L, respectively [33]. The toxicity of these commonly used acaricides such as abamectin (1.8%), emamectin benzoate (1%) and pyridaben (15%) was significantly higher than thyme oil whose LC_50_ was 762.1 mg/L. However, the acaricidal activity of thyme oil was at the same magnitude as plants and essential oils which have been reported to have the potential to be developed into botanical acaricides. For example, Ding et al. found that the acetone extract of roots of *Phytolacca americana* L. possessed acaricidal activity against carmine spider mite with the LC_50_ of 5539.1 mg/L [4]. The LC_50_ of the crude extract of radix stemonae against carmine spider mite was 633.9 mg/L [34]. Chen et al. screened the acaricidal activity of some plant essential oils against carmine spider mite and found that patchouli oil and zanthoxylum oil had a strong toxicity with their LC_50_ of 5480 mg/L and 7770 mg/L, respectively [35].

It has been demonstrated that plant essential oils often possessed stronger insecticidal activity than any of their individual constituents because of the synergistic and additive effects [20,30]. Our result that thyme oil exhibited stronger toxicity than all its major constituents tested individually was in agreement with that phenomenon. However, in the study of Afshar et al., *H. nodiflorum* oils showed lower insecticidal activities compared to some of their main constituents tested separately [36]. The lower activity of natural oil than its single constituent may be due to the antagonistic interactions between the constituents.

### 3.2. Contribution of Individual Compounds to Overall Activity

Our result showed that the toxicity of natural thyme oil was higher than that of an artificial mixture. The phenomenon that the toxicity of the artificial mixture of essential oil was not as high as the natural essential oil has also been previously proved by many researchers. For example, Jiang et al. found that mortality caused by the natural *L. cubeba* essential oil was significantly higher than that caused by the full artificial mixture of the six constituents [37]. However, there are few exceptions. Kim et al. found that there was no significant difference in the fumigant toxicities between full artificial mixture and commercial essential oils of hyssop oil, majoram oil and *Thymus zygis* oil [38]. As the mortality of natural thyme oil was significantly higher than the full artificial mixture in our study, other compounds, although in minor proportion (2.2%), might also be a contributor to the total toxicity of the oil. Further studies need to be done to focus on their identification and contribution to the total toxicity of the oil.

As shown by our results, the toxicity level of the most toxic constituent (M1) or the whole mixture of these active constituents (M1 + M2) was not as high as expected. The blend of inactive constituents (M3) did not cause any mortality. However, combination of M3 with M1, or M2 or M1 + M2, showed significantly greater toxicity than that without the inactive constituents. Only when the blends of active constituents were mixed with inactive ones, did the toxicity of artificial mixture reach the highest level. This indicated that the inactive constituents may have some synergistic effect on active constituents, and, although not active individually, their presence was necessary to achieve full toxicity. Similarly, Pavela et al. found that the toxicity of essential oil can be remarkably enhanced by its minor substances or substances that had no or minimal activity when tested alone. Specifically, borneol and camphor are substances that caused only low mortality when used alone, but both of them provided a high synergistic potential [39]. The fact that the insecticidal activity of an essential oil is not always determined by its main molecules, but reflects the molecule interactions of all constituents insect physiology and insect behavior has also been confirmed by many other studies [20,36,40].

It should be emphasized that we cannot unequivocally say a compound has low toxicity in the case that the elimination of this compound does not affect the overall mortality. The concentration of the compound should also be considered. In the research of Afshar et al., dillapiol was one constituent of *H. nodiflorum* essential oil and had a lower LD_50_ value (97.6 μg/insect) than natural oil (128.4 μg/insect). However, its elimination from the artificial oil did not affect the mortality because of its relatively low concentration (9.4%) [37]. Another study showed that a dillapiole-rich essential oil possessed significant insecticidal activity [41]. Such a phenomenon also existed in our study, but was not so obvious as in the study of Afshar et al. [37]. According to the results of Section 2.2, the toxicity of carvacrol was about the same magnitude as that of terpinene, linalool and *p*-cymene, but whereas the component elimination results indicated that terpinene, linalool and *p*-cymene were moderately active constituents, carvacrol was an inactive constituent. The reason why the elimination of carvacrol did not significantly affect the total toxicity may be also related to the low proportion of carvacrol (2.5%) in thyme oil.

### 3.3. Synergy among Constituents

Similar to our results, the synergistic effect between aromatic compounds has been observed and discussed in a few publications. For example, the binary mixtures of three active constituents (thymol, *p*-cymene and linalool) of thyme oil in our study were proved to have synergistic toxicity against the third instar larvae of tobacco cutworm [42]. Hummelbrunner and Isman examined the synergistic effect of four terpenes on acute toxicity to *Spodoptera litura* larvae and the most significant synergistic effect was found for the combination of thymol with trans-anethole [43].

These synergistic effects of essential oil constituents are possibly useful in two aspects. Firstly, they highlight the importance of using natural essential oils for pest control, rather than separating or synthesizing their major constituents, as the natural essential oils might be more efficacious and pestss may develop resistance slowly to pesticide based on only a couple of active compounds. Secondly, understanding the contribution of each constituent in the toxicity of the oil and the synergy between them could promote the development of more efficacious pesticides based on mixtures of monoterpenoids that do not exist in nature.

Synergistic insecticidal activities existed in many cases, not only between constituents of essential oils, but also between monoterpenoid compounds [21], essential oils [19], and synthetic insecticides [44]. In addition, some essential oils or its major constituents can also be used in combination with synthetic pesticides. This can enhance the insecticidal activities of conventional pesticides, while, at the same time, reduce the development of resistance. The mixture of four kinds of essential oils can remarkably increase the toxicity of abamectin, fenpropathrin, matrine and propargite against pest mites [45]. It has been reported that zanthoxylum oil, artemisia oil, thuja leaf oil and *Artemisia annua* oil can enhance the toxicity of fipronil against *Plutella xylostella* at a low concentration of 1 μL/mL [46]. It should be noted that although plant essential oils can act as synergists of insecticides, antagonistic action against certain pesticides may also occur. Tong and Bloomquist evaluated the effects of 14 plant essential oils on the toxicity of carbaryl and permethrin against *Aedes aegypti* and found that six essential oils had significant synergistic effect on carbaryl. Paradoxically, all of the 14 essential oils decreased the toxicity of permethrin [47]. Blends of *S. montana* essential oil and *P. nigra* essential oil (1:1) were also found to have an antagonistic effect [48]. These findings reminded us that not only the synergism but also the antagonism should be taken into account when we use essential oils to control pests. Whether thyme oil and its major constituents have the synergistic or antagonistic effect on other essential oils and conventional pesticides is the focus of our future studies.

## 4. Materials and Methods

### 4.1. Chemicals

Commercial thyme oil and all individual standard compounds were obtained from Guangzhou Hanson Fragrance Co. Ltd., (Guangzhou, China). The purities of these compounds varied from 95% to 99.5% (thyme oil (≥99.5%), benzoic acid (99.5%), α-pinene (99%), *p*-cymene (98%), terpinene (≥95%), linalool (98%), thymol (98%), carvacrol (99%) and ethyl gallate (96%)). All of them were kept at 4 °C. Tween-80 (laboratory purity) used as an emulsifying agent was purchased from Jiangsu Haian Petrochemical Plant (Jiangsu, China). Acetone of analytical grade was obtained from Chengdu Kelong Chemical Reagents Co., (Chengdu, China). *n*-hexane of HPLC grade was purchased from Merck (Darmstadt, Germany).

### 4.2. Test Mites

Adult carmine spider mites (*Tetranychus cinnabarinus (Boisduval)*) that were used for bioassay had been raised in the laboratory for over 10 years without any pesticide exposure. The mites were reared on cowpea seedlings *(Vigna unguiculata)* in a growth chamber at 26 ± 1 °C and 75–80% relative humidity under a 14 h light/10 h dark cycle.

### 4.3. Bioassays

The slide-dip method recommended by Food and Agriculture Organization of the United Nations (FAO) [49] and the Chinese Agricultural Standard NYT 1154.12-2008 (Guideline for laboratory bioassay of pesticides, Part 12: Slide-dip method immersion) [50] was used for bioassays. The adult mites was picked up with a fine brush and the dorsal surface of the mite was attached to one end of a glass slide by a double-sided adhesive tape. Each slide contained approximate 40 mites and was maintained at 25 ± 1 °C and 65–80% relative humidity for 4 h, following which the mites were examined under a binocular microscope to remove any dead or unanimated ones, thereby leaving only the vivid adults as the original number. The test compounds were diluted to different concentrations by water with 9% acetone and 1% Tween-80. Then each slide with mites was dipped into the given solution for 3 s. The mites dipped into water with 9% acetone and 1% Tween-80 served as controls. The extra solution at the edge of the slides was carefully absorbed by filter paper. Mite mortality was determined under a binocular microscope 24 h after treatment based on movement of the legs or abdomen when prodded by a fine brush. All treatments were replicated 4 times. The mortality had to be corrected by applying Abbott’s formula as follows [49,50]:

(1) $$\text{Corrected mortality}=\frac{\mathrm{test}\;\mathrm{mortality}\;\%-\mathrm{control}\;\mathrm{mortality}\;\%}{100-\mathrm{control}\;\mathrm{mortality}\;\%}\times 100$$

If the mortality in control were above 10%, the bioassay test would be nullified and repeated. The slide-dip method described above was used for all bioassays in Section 2.2, Section 2.3 and Section 2.4.

### 4.4. Gas Chromatography-Mass Spectrometry Analysis

The chemical composition of thyme oil was analyzed by an Agilent 6890A/5973N gas chromatograph mass spectrometer (Agilent Technologies, Santa Clara, CA, USA), operating on electron impact (EI) mode (equipped with a HP 5MS capillary column of 30 m length × 0.25 mm diameter × 0.25 μm film thickness). The initial temperature was set at 50 °C for 2 min, and then gradually increased to 280 °C at a rate of 15 °C/min and held for 5 min. The injector temperature was maintained at 270 °C. 1 μL sample (diluted to 1% with *n*-hexane) was injected with a split ratio of 1:40. Helium was used as the carrier gas with the flow rate of 1 mL/min. EI mode was set as 70 eV. Spectra were scanned from 40 to 550 *m*/*z* at 2 scans s^−1^. Major constituents were identified by comparing their retention indices with those reported in literatures. A series of standard solution of C_5_–C_36_-alkanes were used to obtain the retention indices of compounds under the same operating conditions. Further identification was conducted by comparing their mass spectra with the Wiley 09 (Wiley, New York, NY, USA) and NIST 08 MS libraries (Standard Reference Data, Gaithersburg, MD, USA) or with mass spectra from literature [51]. Component relative percentages were calculated based on GC peak areas without using correction factors.

### 4.5. Comparative Toxicities of Individual Thyme Oil Constitutions

The toxicity of natural thyme oil and each individual constituent was determined at two different concentrations. The first concentration was determined by multiplying 2500 mg/L by the proportion of that individual constituent. The second concentration was 2500 mg/L at which all individual constituents were tested. To further compare the toxicity of thyme oil and thymol, the toxicity regression lines of thyme oil and thymol were calculated using five different concentrations.

In order to identify the contribution of each constituent to the overall toxicity of thyme oil, a series of artificial essential oils were made either as the full mixture or as a mixture each lacking one of the eight major constituents (proportion > 1%). The artificial blends were prepared according to the composition in natural thyme oil indicated by GC-MS and tested at the concentration at which the natural thyme oil caused 95% mortality. The amount of missing compound was replaced by acetone. The types and concentrations of constituents were confirmed by re-analyzing the artificial thyme oil with the same condition mentioned in Section 4.4. Mortalities of the complete and incomplete blends were compared with that of natural thyme oil. Finally, constituents that contributed to the toxicity of the oil were taken as putatively active constituents while constituents that did not affect the overall toxicity were taken as putatively inactive constituents. Toxicities of both active and inactive constituent blend were then compared.

### 4.6. Combined Toxic Effect of the Active Compounds

The combined actions of different binary combination of five active constituents were assessed to determine the types of interaction between them. The binary mixtures of these individual compounds were prepared at the concentration at which the individual compound caused about 30% mortality with the ratio of 1:1 (volume). The co-toxicity factor (CTF) taken as a criterion for evaluating of the combined toxic effect was calculated as follows:

(2) $$\mathrm{CTF}=\frac{\mathrm{OM}\;-\;\mathrm{EM}}{\mathrm{EM}}\times 100$$

where OM is the observed mortality (%) of the combination, and EM (expected mortality) is the sum of mortality (%) caused by each compound of the combination when the compound is tested individually. A positive factor of +20 or higher indicates a synergy effect, a negative factor of −20 or lower means an antagonism effect, and values between −20 and +20 imply an additive effect [52,53].

### 4.7. Statistical Analysis

Mortality data were analyzed using the SPSS software version 18.0 (IBM, Armonk, NY, USA) for analysis of variance (ANOVA). Tukey’s test was used to compare means. Probit analysis was used to determine the toxicity regression, LC_50_ and the corresponding 95% confidence intervals, using the EPA Probit Analysis Program version 1.5 (EPA, Washington, DC, USA).

## Figures and Tables

**Table 1:** Major constituents of thyme oil and mortality caused by thyme oil and its individual constituents.

Constituent	Retention Time (min)	RI ^a^	Proportion (%, *w/w*)	Mortality (%)	
				A	B
Thyme oil	—	—	—	95.5 ± 3.3 A	95.5 ± 3.3 a
α-Thujene	4.1	906	0.3	—	—
Benzoic acid	4.3	928	1.2	0 D	19.5 ± 4.3 d
α-Pinene	5.2	932	1.4	0 D	20.6 ± 2.4 d
Camphene	5.7	943	0.2	—	—
*p*-Cymene	6.8	1018	30.8	11.9 ± 5.1 C	32.6 ± 3.4 c
Terpinene	7.4	1065	15.6	6.6 ± 3.9 C	34.5 ± 4.5 c
Camphor	8.3	1096	0.3	—	—
Linalool	8.4	1102	9.4	3.6 ± 2.9 C	39.8 ± 3.7 c
Borneol	9.6	1165	0.2	—	—
Thymol	11.4	1294	34.6	42.5 ± 4.8 B	78.6 ± 4.5 b
Carvacrol	15.4	1306	2.5	0 D	32.5 ± 3.4 c
Ethyl gallate	28.3	1430	1.3	0 D	13.3 ± 2.4 d
Others	—	—	2.2	—	—

RI ^a^: retention index as determined on a HP-5MS column using the homologous series of *n*-alkanes (C5–C36). Column A: Thyme oil was tested at the concentration at which it caused 95% mortality (2500 mg/L). Major individual constituents (proportion > 1%) were tested at the concentration determined by multiplying 2500 mg/L by the proportion of that individual constituent. Column B: Thyme oil and all individual constituents were tested at the same concentration (2500 mg/L). Data are the mean of four replicates (40 mites per replicate) and are represented as mean ± standard deviation. Means in each column followed by different letters are significantly different (Tukey’s test, *p* < 0.05).The mortalities of controls are from 0 to 5%. —: Untested.

**Table 2:** Toxicity regression lines of thyme oil and thymol based on mortality after 24 h of treatment.

Test Reagents	Regression Equation	LC_50_ (mg/L)	95% Confidence Limit (mg/L)
Thyme oil	Y = 0.4632 + 1.5742x	762.1	621.7–934.1
Thymol	Y = 1.9906 + 2.2746x	1183.9	968.1–1447.9

Five concentrations were used to determine the toxicity regression lines (200, 400, 800, 1600 and 3200 mg/L for thyme oil and 250, 500, 1000, 2000 and 4000 mg/L for thymol). *n* = 4 replicates (40 mites per replicate) per concentration.

**Table 3:** Synergistic effect of binary mixtures of five different acaricidal compounds in thyme oil.

The Combinations	OM ^a^	EM ^b^	CTF
Thymol + Terpinene	79.4 ± 5.4	63.1	25.8
Thymol + Carvacrol	65.7 ± 6.3	57.0	15.3
Thymol + Linalool	74.3 ± 6.5	61.7	20.4
Terpinene + Linalool	69.6 ± 6.2	59.9	16.2
Terpinene + Carvacrol	59.9 ± 5.2	53.3	12.4
Thymol + *p*-Cymene	65.8 ± 5.5	58.8	11.9
Linalool + *p*-Cymene	61.5 ± 5.6	56.8	8.3
Terpinene + *p*-Cymene	57.2 ± 5.8	56.2	1.8
*p*-Cymene + Carvacrol	44.6 ± 5.3	54.6	−18.3
Linalool + Carvacrol	42.3 ± 5.7	58.6	−27.8

A binary mixture of these individual compounds was prepared at the concentration at which the individual compound caused about 30% mortality with the ratio of 1:1 (volume). Data of OM are the mean of four replicates (40 mites per replicate) and are represented as mean ± standard deviation. The mortalities of controls are from 0 to 5%. OM ^a^: observed mortality; EM ^b^: expected mortality. CTF: co-toxicity factor.
